# Smokers’ Decision Making: More than Mere Risk Taking

**DOI:** 10.1371/journal.pone.0068064

**Published:** 2013-07-02

**Authors:** Eyal Ert, Eldad Yechiam, Olga Arshavsky

**Affiliations:** 1 Department of Agricultural Economics and Management, Faculty of Agriculture Food and Environment, The Hebrew University of Jerusalem, Rehovot, Israel; 2 Faculty of Industrial Engineering and Management, Technion, Haifa, Israel; The University of Auckland, New Zealand

## Abstract

The fact that smoking is bad for people’s health has become common knowledge, yet a substantial amount of people still smoke. Previous studies that sought to better understand this phenomenon have found that smoking is associated with the tendency to take risk in other areas of life as well. The current paper explores factors that may underlie this tendency. An experimental analysis shows that smokers are more easily tempted by immediate high rewards compared to nonsmokers. Thus the salience of risky alternatives that produce large rewards most of the time can direct smokers to make bad choices even in an abstract situation such as the Iowa Gambling Task. These findings suggest that the risk taking behavior associated with smoking is not related to the mere pursuit of rewards but rather reflects a tendency to yield to immediate temptation.

## Introduction

Tobacco use, particularly cigarette smoking, is a major preventable cause of premature death and disease. Globally, nearly five million persons die every year from tobacco-related illnesses. In the United States alone smoking is responsible for nearly 443,000 deaths each year (about 18% of total US deaths, [1]). Nevertheless, a substantial amount of people are still smoking. The 2011 National Health Interview Survey (NHIS) indicated that approximately 19.3% of US adults are cigarette smokers [2]. In Israel, where the current study was conducted, about 23% of the adult population smokes cigarettes [3].

Given the clear negative consequences of smoking, the substantial number of smokers is puzzling. This puzzle has led scholars to search for potential traits associated with smoking, in the hope of better understanding the decision to smoke. A natural “suspect” is smokers’ attitude towards risk. There is evidence that smokers are well aware of the risks of smoking ([4]; though Slovic [5] suggests that they do not interpret them correctly). In fact, some studies showed that smokers are even more informed of the risks of smoking than nonsmokers [6]. These findings may suggest that smokers are generally risk insensitive. In line with this reasoning, studies have indicated that compared to nonsmokers, smokers are more likely to partake in a variety of risky behaviors other than tobacco use. For example, smokers tend to be more involved in traffic accidents [7], are less likely to wear seatbelts [8], [9], and are more likely to engage in risky sexual behavior [10]. In addition, women smokers report 12–15% lower rates of mammography checks than nonsmokers [11].

These surprising findings have motivated more controlled examinations for possible differences between smokers and nonsmokers. One interesting area of research focuses on differences in personality, and has shown that smoking is associated with impulsivity [12], [13], psychoticism [13], defensive optimism [14], [15], and sensation seeking [16].

A complementary line of research focuses on behavioral measures of risk taking in controlled laboratory tasks. A commonly used task in this context is the Iowa Gambling Task (IGT; see [17], [18]) in which individuals choose repeatedly between four decks of cards, associated with different payoff distributions, for an unspecified number of trials. Two of these payoff distributions are characterized by relatively higher risk (larger gains and losses) compared to the other two decks, as well as by greater immediate benefits but lower long term outcomes (see Table 1). Typically, individuals with brain lesions in areas affecting decision making were found to take more risk and perform more poorly in this task [17], [18]. The IGT was also found to distinguish chronic cannabis and cocaine abusers from non drug-abusing controls (e.g., [19], [20]). However, in a recent study, Lejuez et al. [21] used the IGT to compare smokers to nonsmokers, but did not find significant differences between the two groups (see also Aklin et al. [22]). These results suggest that smokers’ ability to learn the possible harmful consequences of risky behavior is not impaired [4]. Yet they still leave open the question of why smokers exhibit risky behavior in different domains as noted above.

**Table 1:** The schedule of rewards and penalties in the four decks of cards of the IGT, as introduced in [20]: The first block of 10 selections from each of the decks (out of 6 blocks).

Card	Deck Dis50Win $ 100 every trial	Deck Dis90Win $ 100 every trial	Deck Adv50Win $50 every trial	Deck Adv90Win $50 every trial
1				
2				
3	-$150		-$50	
4				
5	-$300		-$50	
6				
7	-$200		-$50	
8				
9	-$250	-$1250	-$50	
10	-$350		-$50	-$250
Average loss	-$125	-$125	-$25	-$25
Frequency of loss	0.5	0.1	0.5	0.1
Average gain *	$100	$100	$50	$50
Average gain – loss	-$25	-$25	$25	$25

Note: The average gains and losses are across all 10 selections. The gains on each trial range from $80-$120 for decks Dis50 and Dis90 (normally distributed in discrete steps of $10) and from $40-$60 for decks Adv50 and Adv90 (normally distributed in discrete steps of $5). In addition, the difference between the decks’ expected values increases with time. That is, after each 10 selections the average gain for the disadvantageous decks increases by $10 while the average loss increases by $25. At the same time, the average gain for the advantageous decks increases by $5 while the average loss increases by only $2.5.

The main objective of this paper is to better understand the psychological constructs that contribute to smokers’ risk taking. We suggest that it is not risk taking per se that drives the difference between smokers and nonsmokers’ decision making. Rather, we argue that the potential link between smoking and risk taking lies in the person’s ability to resist temptations. Indeed, previous research has suggested a close relation between visceral urges and smoking [23]. In a series of studies, Baumeister and his colleagues have shown that smokers tend to exhibit poorer self control than nonsmokers [24], [25]. Additionally, training of self-control seemed to improve people’s ability to give up smoking [26].

In the context of decision making, we propose a condition where risk taking can be facilitated by poor self-control. Selecting alternatives that result in poor immediate outcomes but high long term outcomes requires the exercise of self control [27]. Hence, when all rewards are transparent poor self control facilitates the preference for a risky alternative that frequently produces the best reward, even if its expected value is negative. Using two modified versions of the IGT, we predicted that smokers’ risk taking is driven by their tendency to be more easily tempted by common favorable outcomes, rather than by a general tendency towards risk taking.

## Methods

### Ethics Statement

Before the beginning of the study the participants were provided with a written statement, which specified the research procedure. Participants gave their informed consent both verbally and by clicking a response button that asked for their approval to participate in the experiment. As anonymity was vital for the success of the study, this procedure ensured that no record would link their identity to this research. The experiment and its consent procedure were approved by the Technion Ethics committee for Human Studies in the Behavioral Sciences.

### Task Description

As presented in Table 1, our modified version of the IGT involves choices between four different alternatives in each of 60 trials. The IGT includes two disadvantageous decks that yield negative return in the long run: Dis50 and Dis90. The latter deck (Dis90), however, also produces the highest payoff in most trials, and is associated with the smallest probability of losing. Thus, it presumably takes a certain amount of self-control to consistently make the decision of *not* selecting this option (indeed, most individuals are attracted to options with high common payoffs and rare negative outcomes [28]–[30]). Additionally, in our version of the IGT, the outcomes of all (chosen and unchosen) decks are revealed after each trial. The use of such “foregone payoffs” was expected to further increase the self control challenge of avoiding deck Dis90, as it highlights its relative advantage over all other decks in a typical trial (e.g., [28], [31], [32]). Previously, the administration of foregone payoffs was found to increase risk taking among high functioning drug abusers [28].

In order to further evaluate this assertion we administered a second task where risky choices were not associated with common (or uncommon) events. This task, referred to as EGT (Equal-probability Gambling Task) is described in Table 2 (it is a variant of the task used by Lane et al. [33]). Since there is no alternative associated with the best common outcome in this task, the tendency to select the best recent outcome should not affect risk taking. Thus, we expected no differences in risk taking between smokers and nonsmokers in the EGT.

**Table 2:** The schedule of rewards and penalties in the four decks of cards of the EGT.

Card	Safe/Risky	Alternative: Payoff
Deck Dis50a	Risky	Win or lose $50, $100, $150, $200 with equal chances
Deck Dis50b	Risky	Win or lose $50, $100, $150, $200 with equal chances
Deck Adv50a	Safe	win $20 for sure
Deck Adv50b	Safe	win $20 for sure

Note: The payoffs of the risky decks are drawn randomly from the uniform distribution (-200, -150, -100, -50, 0, 50, 100, 150, 200). The payoffs are drawn once before the experimental session and then are fixed to be identical to all participants.

### Participants

One hundred Technion students (51 males and 49 females) participated in the experiment. Participants were paid NIS 30 (about $7.50) for showing-up plus the monetary bonus they had earned in association with their performance. They earned on average about NIS 55.

### Apparatus and Procedure

Before the beginning of the study participants were instructed about the research procedure and gave their informed consent. Then, each participant was presented with two behavioral tasks: (a) the modified IGT and (b) the EGT (presented in Table 2). The modified IGT involved choices between four decks of cards. The task included 60 trials. Payoffs were drawn from six decks of 10 cards, as described in Table 1. In each consecutive block the differences between advantageous and disadvantageous decks increased linearly. Participants were given written instructions identical to those provided in [28]. Briefly, participants were told that some decks are worse than others, and they should avoid those decks to win the game. After each selection participants were presented with foregone payoffs, which were displayed on the unselected decks for an unlimited time. Participants transitioned to the next trial by pressing a button labeled “Move to the next choice.” The EGT used the same procedure as the IGT except that its alternatives were associated with different outcomes (see Table 2). The ordering of the two tasks was counterbalanced. Participants were told that their accumulated task payoff would be added to their show-up fee.

After the completion of the behavioral tasks, we used several additional measures. We administered the Eysenck Personality Questionnaire – Revised (EPQ-R-S [34]; Hebrew version, see [35], [36]) to identify possible differences between smokers and nonsmokers’ personality traits. We also ran a computerized version of Set 1 of the Raven Progressive Matrices test [37] to verify that there were no differences in IQ between smokers and the control group.

Participants’ last task was to answer a demographic questionnaire whose main objective was to identify smokers. Specifically, participants were asked whether they smoked, or used to smoke in the past. Those who responded positively were asked how many cigarettes they had smoked per day in the last six months. Thirty three participants (15 males and 17 females) were classified as smokers. Initially, out of 87 people only 20 were identified as smokers. To increase the proportion of smokers in our sample, we added the last 13 people by first delivering a phone interview asking about various habits (including smoking) and then inviting smokers only to the lab. We did not identify any significant differences in behavior between these 13 smokers and the other 20 smokers. The median number of cigarettes smoked per day was 5 and the mean was about 7, so the smokers in our samples can be considered as relatively light smokers. Additionally, seven participants (5 males and 2 females) were identified as past smokers. These past smokers were treated in our main analysis as nonsmokers but we also conducted a secondary analysis to see whether the results change when past smokers are classified as smokers. The data is available at the first author’s website: http://departments.agri.huji.ac.il/economics/teachers/ert_eyal/ publications.htm.

## Results

**Figure 1:**
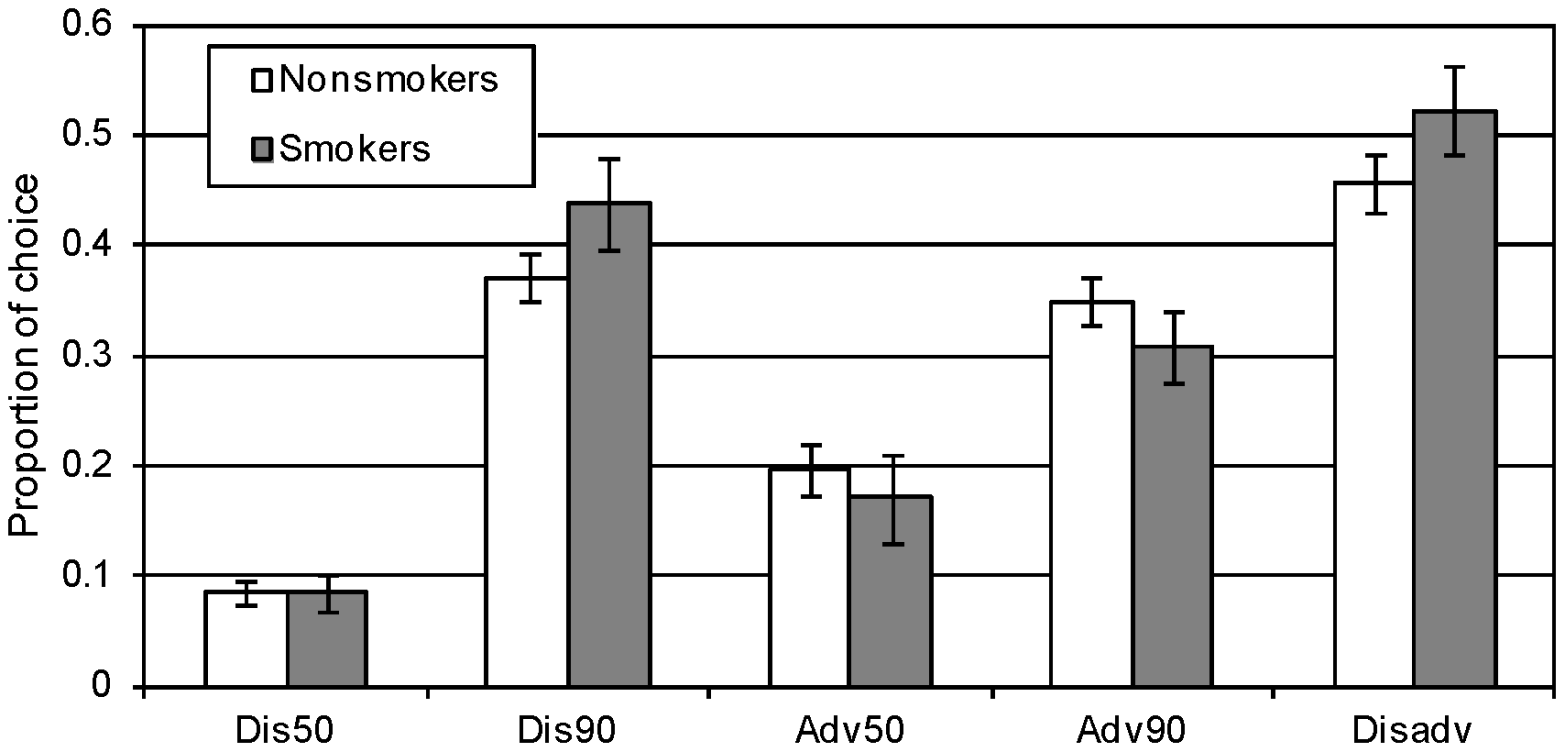
Smokers and nonsmokers’ choices from each of the decks of the modified IGT (average proportions and standard errors). The right column (Disadv) shows the proportion of choices from the disadvantageous decks (Dis50+Dis90).

The choice patterns in the IGT are summarized in Figure 1 which presents the choice proportion from each deck, as well as the proportion of disadvantageous choices across trials. The results show a weak (and insignificant) tendency of smokers to choose more disadvantageously than nonsmokers (Smokers: 0.54, SD  = 0.19; nonsmokers: 0.46, SD  = 0.25; *t*(98)  = 1.49, NS).

**Figure 2:**
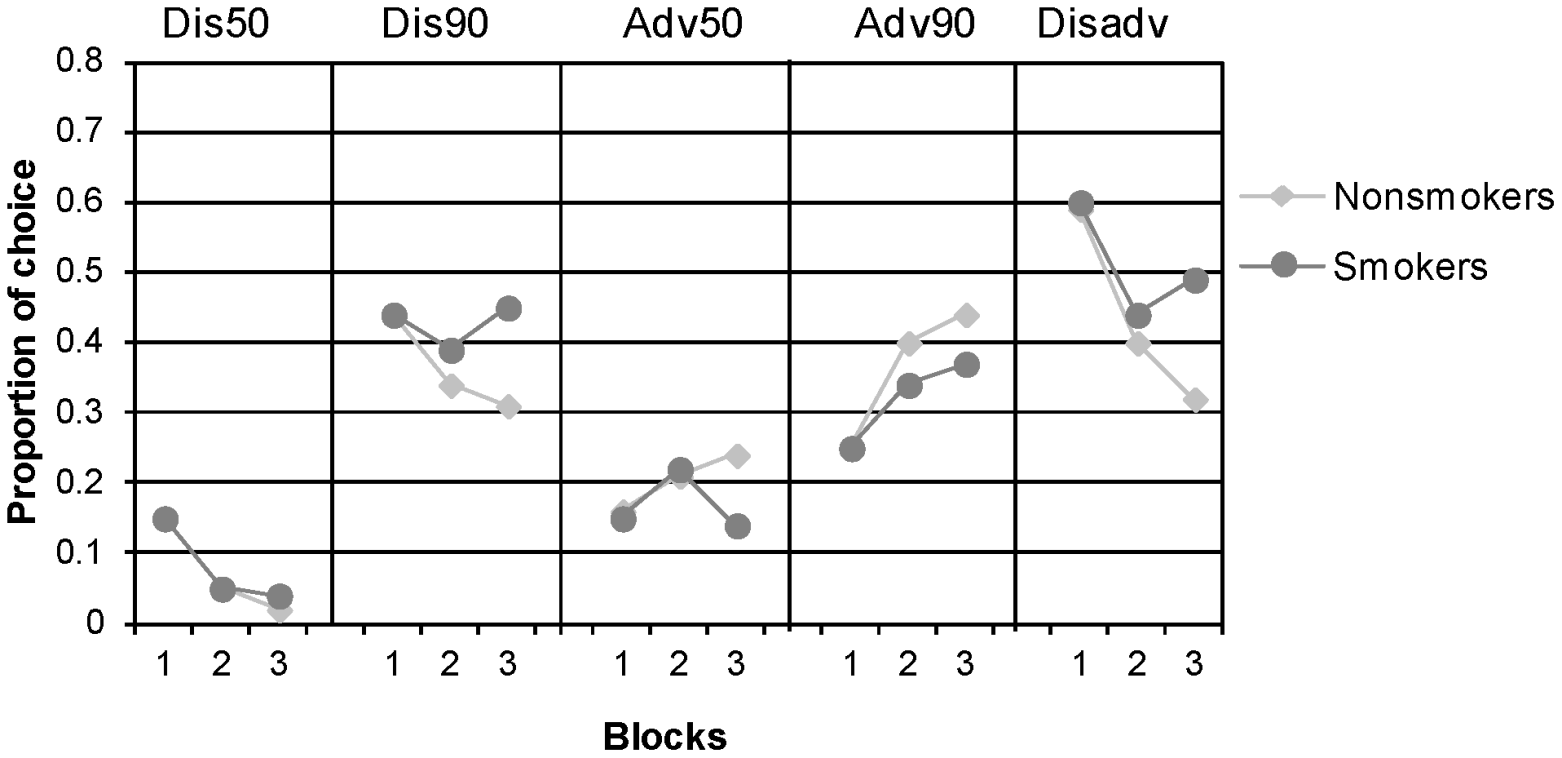
Smokers’ and nonsmokers’ choices as a function of time, by blocks of 20 trials, in the modified IGT (average proportions). The right column (Disadv) shows the proportion of choices from the disadvantageous decks (Dis50+Dis90).

We proceeded to examine whether differences would be found in the learning patterns for individual decks. Figure 2 presents the learning patterns of smokers and nonsmokers for each deck. These patterns were evaluated with a repeated measures analysis of variance with trial block (3 blocks of 20 trials) as a within subject variable, and smoking as a between subject variable. The results revealed that, with experience, smokers increased their selections from Deck Dis90, whereas nonsmokers learned to avoid it, F(2, 180)  = 2.99, *p*  = .05. This pattern of choices led nonsmokers to choose more advantageously with experience, whereas for smokers the learning curve was milder, F(2, 180)  = 2.20, *p*  = .09.

In line with these differences in mean performance, an individual level analysis [36] showed that only 33% of the smokers learned from their experience and improved their advantageous selections in each block (of 20 trials) compared to 54% of the nonsmokers, Z  = 1.97, *p*  = .048.

These results demonstrate that, similarly to high-functioning drug abusers [28], smokers could not resist the temptation of the constantly presented high rewards associated with deck Dis90. Thus, smokers’ preference of high common outcomes has led them to make bad choices in the task. In order to examine a potential mechanism leading to this pattern of selections, we ran a trial by trial analysis that focused on the proportion of choices from the alternative that yielded the best outcome in the most recent trial (a pattern called “chasing” [38]). The analysis revealed that smokers chased the best recent outcome 39% (SD  = 17%) of the time, while nonsmokers chased it only 30% of the time (SD  = 26%). This difference was significant, *t*(98)  = 2.12, *p*  = .04.

**Figure 3:**
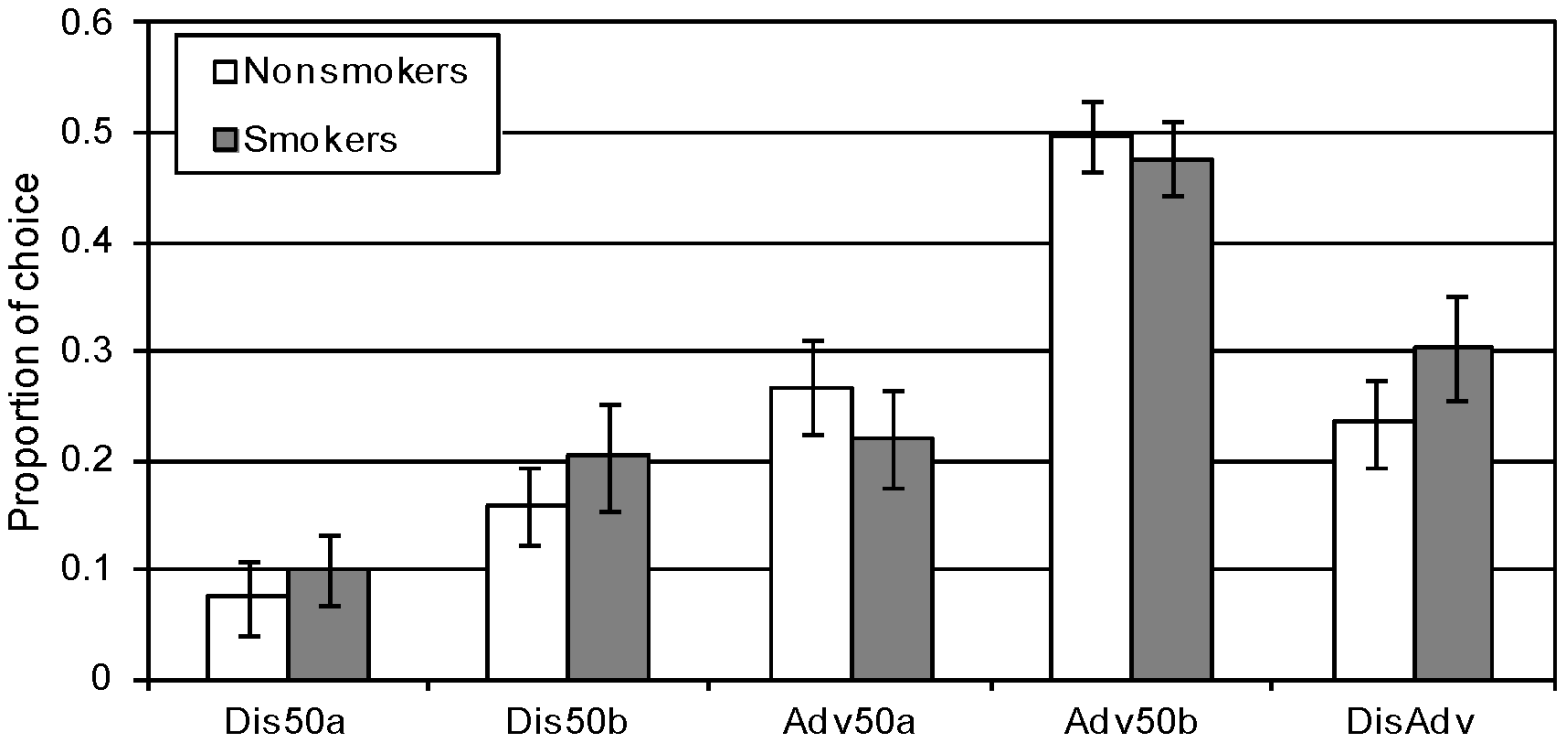
Smokers and nonsmokers choices from each of the decks of the EGT (average proportions and standard errors). The right column (Disadv) shows the proportion of choices from the disadvantageous decks (Dis50a+Dis50b).

**Figure 4:**
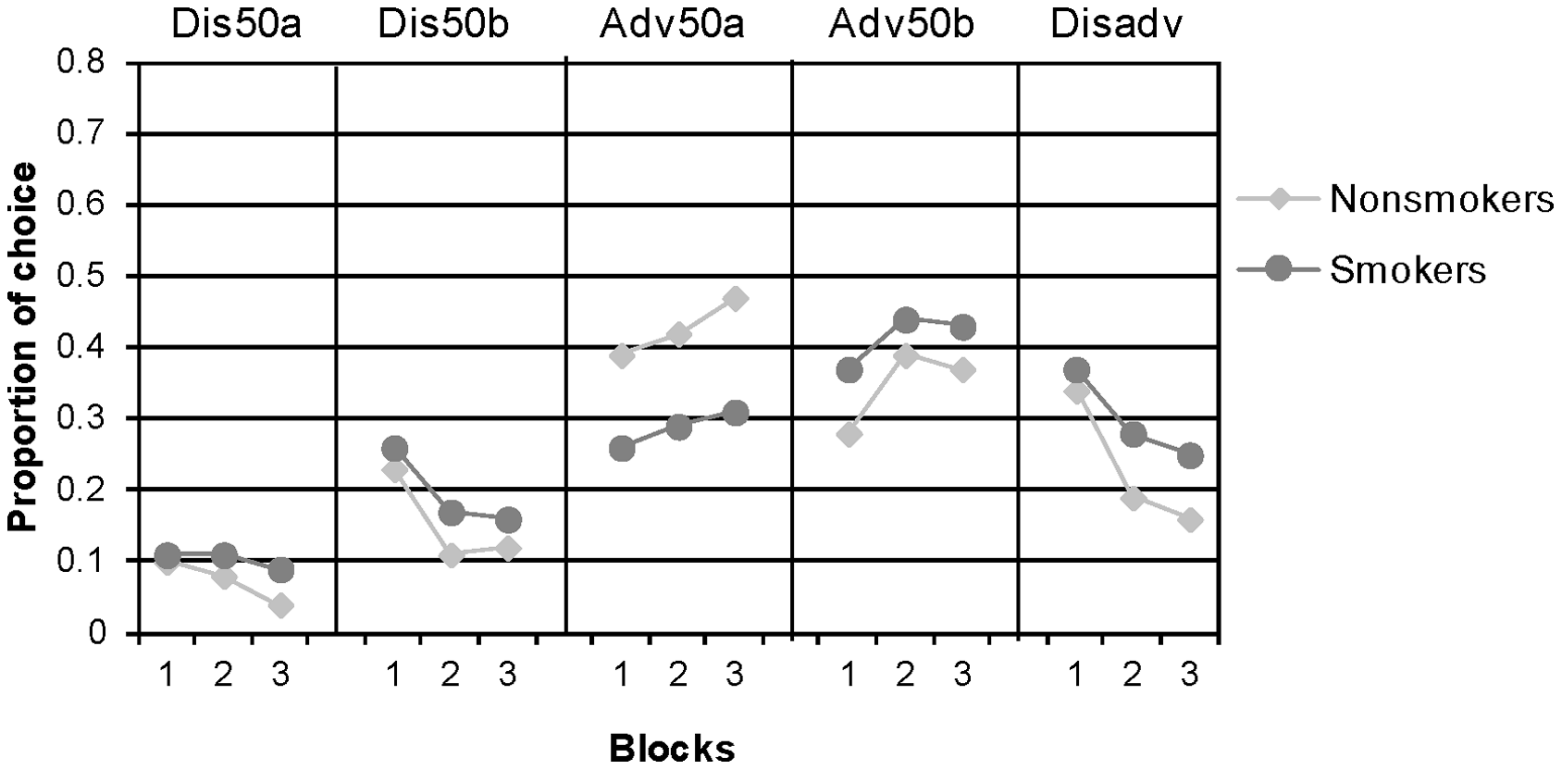
Smokers’ and nonsmokers’ choices as a function of time, by blocks of 20 trials, in the modified EGT (average proportions). The right column (Disadv) shows the proportion of choices from the disadvantageous decks (Dis50a+Dis50b).

The results of the EGT, presented in Figure 3, showed that, similar to the IGT, there was a weak tendency among smokers to choose more disadvantageously than nonsmokers (smokers  = 0.30, SD  = 0.22; nonsmokers  = 0.23, SD  = 0.25; *t*(98)  = 3.11, *p*  = .08). Yet an analysis of the learning patterns, depicted in Figure 4, reveals that, unlike in the IGT, smokers have not become more attracted to the disadvantageous decks in the course of their experience. Both smokers and non- smokers learned to choose advantageously with experience, F(2,196)  = 26.81, p<.001, and there were no significant differences in the learning patterns of the two groups, F(2,98)  = 0.33, NS. An individual level analysis showed that 52% of the smokers learned from their experience and improved their advantageous selections in each block compared to 60% of the nonsmokers, Z  = 0.76, NS.

Interestingly, the trial by trial analysis of chasing suggests that in the EGT as well smokers were more sensitive than nonsmokers to attractive recent outcomes. Smokers chose the alternative that yielded the best outcome in the most recent trial in 40% (SD  = 25%) of the cases while nonsmokers chased the best outcome only in 24% (SD  = 11%) of the trials, t(98)  = 4.55, p<.0001. These results replicate the findings from the IGT: smokers were more attracted to alternatives that produced the best recent outcome. Unlike the IGT, however, no specific alternative is associated with the best typical outcome in the EGT. Thus, no specific alternative benefited from smokers’ tendency to chase the best recent outcomes.

A Pearson correlation analysis between the proportion of advantageous choices in the IGT and the EGT suggested that performance was mildly related in these tasks, r  = .17, p  = .09. Interestingly, the correlation among nonsmokers was somewhat higher (r  = 0.18) than among smokers (r  = 0.06), though the difference was insignificant, Z  = 0.85, NS.

Table 3 presents the data from the demographic questionnaire, the Raven test, and the personality questionnaire. Almost no differences were found between the two groups in the EPQ-R-S. The only significant difference was in the Lie scale, *t*(98)  = 3.32, *p*  = .002, in which smokers’ scores were somewhat lower (Mean  = 1.74, SD  = 3.07) than those of nonsmokers (Mean  = 3.52, SD  = 2.86), suggesting that smokers may be less inclined to make a good impression. The Raven test also indicated similar scores for smokers (Mean  = 85.2%, SD  = 15.0%) and nonsmokers (Mean  = 88.8%, SD  = 12.7%). Thus, the disadvantageous choices of smokers could not be attributed to IQ.

**Table 3:** Demographic and personality questionnaire data for smokers and nonsmokers, and the average number of cigarettes smoked per day in the last six months in the smokers group (standard deviations appear in parenthesis).

Variable	Smokers	Nonsmokers
*Demographics*		
Age	24.18 (2.54)	23.73(2.57)
Education	14.58 (1.15)	14.52 (0.99)
Gender	15 M, 17 F	35 M, 32 F
IQ (Raven)	85.2% (15.0%)	88.8% (12.7%)
*Personality Test (PEN)*		
Psychoticism	3.03 (2.46)	2.43 (1.93)
Extraversion	9.73 (2.85)	9.60 (2.97)
Neuroticism	4.64 (3.66)	3.58(3.49)
Lie Scale	3.52(2.86)*	5.25 (3.07)
Smoking Frequency	7.19(8.21)	

* p<.05.

In all the analyses reported above we considered past smokers (7 people) as nonsmokers. We also repeated our analysis by treating past smokers as part of the smokers group. Interestingly, the group differences in the IGT were strengthened. This observation gives further support to the idea that the decision to smoke might be associated, at least to some degree, with certain behavioral traits.

## Discussion

The objective of the current paper was to better understand the constructs that drive smokers’ risk taking behavior. In the context of decision making, we proposed a condition where risk taking can be facilitated by temptation. Specifically, we referred to the case where a risky alternative yields the best common outcomes. The results supported the assertion that, similarly to high-functioning drug abusers [28], smokers could not resist selecting from an alternative that yielded highly noticeable positive rewards, even though it also produced large infrequent penalties resulting in an overall loss. The results further showed that the exclusion of such alternatives (as in the EGT task) did not eliminate smokers’ tendency to chase the best recent payoffs but did reduce the differences in risk taking between smokers and nonsmokers, particularly after gaining experience with the alternatives.

Note that this pattern was observed even though the typical smokers in our sample were light smokers. A potentially related finding is that smokers in our sample did not differ from their peers in the personality traits of the EPQ-R-S. This latter observation differs from that found in other studies which have observed differences in the P and E scales [13], [39]. Possibly, the difference results from the fact that the current study included mostly (74%) light smokers (<10 cigarettes a day), while previous studies focused on heavier smokers. For example, light smokers accounted for only 39% of the smokers sample in [13]. This assertion is supported by the finding that psychoticism and impulsivity were found to be predictive of the number of cigarettes smoked per day [13].

We believe that the current findings shed new light on previous studies that have demonstrated smokers’ tendency to take risk in various naturally occurring circumstances, such as in driving behavior [9], sexual conduct [10], and preventative care [11]. It is interesting to note that all of these past findings focused on situations where risky behavior implies under-sensitivity to a rare disaster, such as catching a sexually transmitted disease, being involved in an accident, or having an undiagnosed fatal illness. Moreover, in most of these analyses the risky option can be easily compared to the safer one, and appears to be significantly more attractive in the short run.

The results of the current paper suggest that it is not the mere inclination to take risk that drives smokers’ behavior in such situations, but rather their tendency not to engage in choices that are inconvenient (even though they protect the person from a small likelihood of a catastrophe), and to be more easily tempted by the typically pleasurable alternative.

The results also clarify one potential cognitive mechanism leading to the tendency of smokers to focus on typically favorable outcomes: The tendency to respond to the most recent events and to improperly integrate outcomes in the more distant past. This tendency has been argued and shown to be implicated in cocaine and marijuana abuse [19], [20], [40] and the current study is the first to demonstrate it in light tobacco smokers. The existence of this shared cognitive mechanism may suggest commonalities in self control impairments between tobacco smokers and other psychoactive substance abusers. Because our study focused on light smokers, we believe that these observed characteristics of the smokers’ population were associated with the predisposition to smoke, rather than the effect of Nicotine use and dependence. In line with this interpretation, in previous studies light smokers or even individuals who just tried smoking a couple of times had lower self control in a behavioral task than never-smokers [41], [42]. Nevertheless, future studies should use controlled periods of abstinence in order to rule out potential effects of Nicotine addiction on decision style and self control.

In light of this self control account, it is interesting to return to the results of Lejuez et al. [21], [22], who found no differences between smokers and nonsmokers in the simple IGT. The simple IGT does not include forgone payoff information, and thus the favorable outcomes from the risky alternatives are less tempting, which may explain the null results in these studies. Lejuez et al. [21], [22] also found that smokers took more risk than nonsmokers in the Balloon Analogue Risk Task (BART). In this task the participants choose in several trials between inflating a virtual balloon or moving to the next balloon. Each inflation results in an increase of money in a temporary cache, and upon moving to the next balloon the participant earns all the money in this cache. However, the probability of explosion (that leads to losing all temporary earnings) increases exponentially with each inflation. Notice that in this setting taking risk (choosing to inflate) is typically more favorable than acting safely (choosing to stop), and this distinction is quite salient (the decision maker is continually exposed to the positive outcomes of the “inflate” strategy). The current analysis therefore suggests that smokers’ attraction to the typically favorable outcome might drive their risk taking in this task.

The present findings are thus instrumental in clarifying the link between studies of decision making and self control in smokers. The different decision making behavior exhibited by smokers in the current study, as well as in other studies reported above, can be interpreted by the assertion that smokers are consistently exhibiting less self-control; which leads them to make choices that are commonly rewarding but may be risky on occasion.

Our findings also have some implications to policies directed at reducing smoking. Specifically, they imply that manipulations aimed at reducing the convenience of smoking are likely to be useful because they reduce the immediate temptation to which smokers are highly sensitive. An example is the policy of banning smoking not only inside buildings but also at an external perimeter [43]. While originally proposed to address secondary smoking hazards, this policy also reduces the immediate convenience of smoking, and it was found to decrease the rate of adolescent cigarette use [44]. In a similar vein, making smoking areas less observable to non-smokers (e.g., by using non-transparent partitions) is expected to lower the immediate foregone pleasure of smoking. Similar strategies of making smoking less convenient could be enacted at the individual level as part of treatment. For example, the individual could be instructed to make changes to his/her smoking environment that increase the effort implicated in a typical smoking session (e.g., by wrapping cigarette packets in several layers of adhesive tape). Future studies should examine whether manipulations of this type are particularly effective for individuals who are chronically low on self-control.
